# Association between Sleep and Suicidal Ideation in Chinese Undergraduate Students

**DOI:** 10.3390/ijerph192315433

**Published:** 2022-11-22

**Authors:** Ran Wu, Chun-Ying Wang, Feng Wang, Yu-Jing Wang, Hong Zhu, Guang-Hai Wang, Chun-Lei Jiang

**Affiliations:** 1 Counseling and Psychological Services Center, East China Normal University, Shanghai 200062, China; 2 Centre for Psychological Health Education and Counseling, Shanghai University of Medicine & Health Sciences, Shanghai 201318, China; 3 Pediatric Translational Medicine Institution, Shanghai Children’s Medical Center, School of Medicine, Shanghai Jiao Tong University, Shanghai 200127, China; 4 Department of Stress Medicine, Faculty of Psychology, Second Military Medical University, Shanghai 200433, China

**Keywords:** sleep quality, sleep hygiene, insomnia symptoms, suicidal ideation, college students

## Abstract

Suicide is an important global public health issue, which deserves more attention. This study aims to examine the relative independent relationship between suicide ideation and subjective sleep quality, sleep hygiene, and insomnia symptoms in undergraduate students in China. This population-based study included 2379 undergraduate students aged 18–26, randomly recruited from three public universities in Shanghai. The participants completed four questionnaires: the Pittsburgh Sleep Quality Index; Sleep Hygiene Practice Scale; Insomnia Severity Index; and the Symptom Checklist 90 (specifically the depression and anxiety dimensions and Q15-suicide ideation). The results of Spearman’s correlation analysis indicate that poor sleep quality, short sleep duration, poor sleep hygiene, and insomnia symptoms were all associated with suicidal ideation in undergraduate students. However, according to the results of the hierarchical linear regression, no experience of sharing a bedroom at home, poor relationship with roommates, short sleep duration, sleep medicine use, and good daytime function were related to suicidal ideation, after controlling for the symptoms of depression and anxiety, which may be important in the identification of suicidal ideation. Sleep problems are highly discoverable and modifiable, and have a low sense of shame, therefore, sleep interventions for individuals with suicidal ideation and poor sleep quality may be an efficient and effective approach to suicide prevention.

## 1. Introduction

Suicide is the fourth leading cause of death worldwide in youth aged 15–29 years across both sexes [1]. Approximately 180,000 individuals in this age group died by suicide in 2019 [1], creating an immense social burden and pain for survivors. This number does not consider other related behaviors, such as suicide attempts (SA) and suicidal ideation (SI), which threaten the lives of young people. Consequently, suicide prevention and the global reduction of suicide rates are crucial.

The complexity and unpredictability associated with suicide risk factors have hindered suicide prevention and intervention efforts [2]. Previous studies have identified the influencing factors and developed a theoretical framework for suicide. Sleep disturbance, a putative and modifiable risk factor, has the potential to become an intervention tool [3], yet it is less integrated into standardized suicide risk assessment frameworks. This may be due to the fact that, until recently, scholarly attention has been focused on the effect of sleep on suicide, and the conclusions of relevant research have been inconsistent. 

According to the results of Kearns et al. [4], 70% of the research they reviewed reported at least one type of sleep disturbance that significantly predicted SI and behavioral outcomes among youths. Sleep disturbance was also found to increase suicide risk 1.95–2.95 times [5]. However, current studies on the impact of sleep on SI often yield inconsistent results. For example, Nadorff et al. have found that nightmares, but not insomnia symptoms, were related to SI after controlling for symptoms of anxiety, depression, and post-traumatic stress disorder (PTSD) in a sample of college students [6]. However, in another sample of college students, Nadorff and Nazem found that only the duration of insomnia symptoms and nightmares were significantly associated with suicide risk [7]. Contradictory findings have also been reported in more recent studies. For example, a study has reported that short sleep duration (≤6 h) compared to normal sleep duration (7–9 h) was associated with the reporting of a prior SA but not SI [8]. Another study has found that participants with insomnia and short sleep duration were associated with 2.09-fold increased odds of SI [9]. Furthermore, existing researches mainly focus on insomnia symptoms, nightmares and sleep duration, and have paid little attention to the relationship between other aspects of sleep (e.g., sleep medicine use, daytime function, and sleep hygiene) and SI. Taking sleep hygiene as an example, a systematic review has reinforced the notion that there is a strong association between sleep hygiene and depression or depressive symptoms [10]. For example, it has been shown that dysfunctional beliefs about sleep can predict SI in adolescents aged 12–17 [11]. However, there is no empirical evidence to suggest that the sleep hygiene of young adults is related to suicidality.

This study aims to enrich existing knowledge regarding the relationship between sleep and suicidality among young people in China. We hypothesize that college students without SI experience better sleep quality and sleep hygiene and reduced insomnia than those with SI. Furthermore, insomnia symptoms, poor sleep quality, and poor sleep hygiene will be significantly related to SI. Therefore, this study focused on sleep disturbance, including poor sleep quality (e.g., long sleep latency, insufficient sleep, low sleep efficiency, sleep medicine use, and daytime dysfunction), poor sleep hygiene (e.g., sleep time arrangement, arousal-related behaviors, poor eating habits before bed, poor sleep environment), and insomnia symptoms. With Chinese undergraduate students as participants, we investigated the relationship between the abovementioned sleep disturbance and SI independently from depression and anxiety symptoms.

## 2. Materials and Methods

### 2.1. Participants and Recruitment

This study was approved by the University Committee on Human Research Protection, East China Normal University (HR 190-2019). We conducted our study across three public universities in Shanghai, China, during July to November 2020. We randomly selected one science major and one liberal arts major from each grade from freshmen to seniors of each university. We randomly distributed recruitment posters to 150 students in each selected major, utilizing WeChat. A total of 3600 posters were distributed and 2434 students agreed to participate in the study. 

Our inclusion criteria were as follows: the participants had to be (1) aged 18 years or older; (2) full-time undergraduate students from one of the three universities chosen; (3) not in need of suicidal crisis intervention; and (4) willing to participate in the study voluntarily. The exclusion criteria were as follows: (1) experienced a major life event (e.g., bereavement) or significant mood fluctuations within the past month; (2) mother tongue is not Chinese; and (3) unable to complete all questionnaires. Approximately 2379 of the participants completed the questionnaires (39 participants withdraw from the study due to scheduling problems, and 16 participants did not complete all the items). The participants signed informed consent forms online before completing the questionnaire, which emphasized that participation was voluntary, and that they could withdraw from the study at any time. 

### 2.2. Measure

#### 2.2.1. Suicidal Ideation and Emotional Health

The Symptom Checklist 90 (SCL-90) was finalized by Derogatis in 1975 [12]. This questionnaire measures a range of psychological and psychiatric symptoms. It comprises 90 items and evaluates nine symptomatic dimensions. In this study, Q15 (thoughts of ending life) was used to evaluate the SI of participants. The depression and anxiety subscales were used to assess depression and anxiety symptoms. All items are scored on a 5 point scale, with average scores of both subscales ranging from 1–5 points. An item with score ≥ 2 or any factor with a score that exceeds two points indicates that the participant has positive symptoms for the item or factor. The Chinese version of the SCL-90 has been shown to have good reliability and validity [13]. Cronbach’s α in this study is 0.98.

#### 2.2.2. Sleep

The Pittsburgh Sleep Quality Index (PSQI) is a self-report questionnaire that evaluates subjective sleep quality over a period of one month [14]. The scale comprises 19 items divided into seven subscales, namely sleep quality, sleep latency, sleep duration, habitual sleep efficiency, sleep disturbances, use of sleep medication, and daytime function. Each item is scored on a scale ranging from 0 to 3. With regard to the sleep duration subscale, only the adequacy of sleep is classified, and the problem of excessive sleep cannot be investigated. The total score ranges from 0 to 21, with a higher score indicating worse sleep quality. Among the Chinese population, a score of more than seven generally indicates poor sleep quality [15,16]. We utilized the same threshold in our study. The PSQI has been shown to have high reliability, and construct validity. The Cronbach’s α of the Chinese version of the PSQI is 0.84 [15]. Cronbach’s α in this study is 0.71.

The Sleep Hygiene Practice Scale (SHPS) was compiled by Jian-Ming Yang [17]. The scale is utilized to investigate the behavior and sleep-related habits of participants from four dimensions: sleep time arrangement, arousal-related behaviors, poor eating habits before bed, and poor sleep environment. The scale contains a total of 30 items, each of which is rated on a scale from 1 = “never” to 6 = “always”, and the total scale score ranges from 30–180. The higher the score, the less scientific the sleep habits. The SHPS has been shown to have good reliability and validity. Cronbach’s α in this study is 0.88.

The Insomnia Severity Index (ISI) was compiled by Morin in 1993. It is a self-assessment questionnaire consisting of seven items and is used to evaluate the severity of an individuals’ subjective insomnia [18]. The items are measured on a five-point Likert scale from 0 to 4. The items relate to the typical symptoms and impact of insomnia, including difficulty in falling asleep, difficulty in remaining asleep, early waking, and sleep satisfaction. Studies have shown that the scale has good internal consistency reliability (Cronbach’s α = 0.74) and is also correlated with patient sleep quality measured by polysomnography (r = 0.32–0.55) [19]. The total score of the scale ranges from 0–28 points and is used to assess the severity of insomnia in patients, with higher scores representing more severe insomnia. According to the total score of the scale, the degree of insomnia can be divided into no insomnia (0–7 points), mild insomnia (8–14 points), moderate insomnia (15–21 points), and severe insomnia (22–28 points). Cronbach’s α in this study is 0.85.

#### 2.2.3. Demographic Characteristics

Data in respect of the participants’ demographic characteristics such as sex, age, level of education, major, and school, were collected.

Questions included whether students have separate bedrooms and beds in their own homes, whether they have siblings, how much time they spend on their mobile phones in bed before sleep, whether they have the same sleeping time as their roommates, whether they have a harmonious relationship with their roommates, and whether they ask for help because of sleep problems.

### 2.3. Statistical Analysis

The Kolmogorov–Smirnov test was conducted to test the normality for the variables used in this study. The results showed that none of the variables met the assumption of normality (all *p* < 0.05). Descriptive statistics (median and interquartile range [IQR]) were used to examine the characteristics of the sample population. All listed variables of participants with or without SI were tested by the Mann–Whitney U test to investigate the differences in sleeping habits, sleep quality, and emotional symptoms between participants with or without SI. Spearman’s correlation analysis was then applied to examine the interrelations between all key variables in the study. Finally, hierarchical linear regression was utilized to examine the predictors of SI. In the first step of the regression models, demographic covariates were entered (i.e., sex, age, share bedroom at home, sleeping schedules consistent with roommates, and relationship with roommates). In the second step, key variables of sleep quality, sleep hygiene, and insomnia symptoms were entered. In the third step, depressive and anxiety symptoms were entered.

IBM SPSS Statistics (version 23.0) was used for all statistical analyses. The *p* values < 0.05 were considered to be statistically significant.

## 3. Results

### 3.1. Sample Characteristics

A total of 2379 Chinese college students completed all the questionnaires and were included in the analysis. The students were aged 18–26 years. The average age of the participants was 19.90 years (SD = 1.13). The demographic characteristics of the participants are summarized in Table 1. 

### 3.2. Characteristics of Sleep and Emotional Symptoms with or without SI

Descriptive data and outcomes of the Mann–Whitney U test for all of the key variables for all participants and participants with/without SI are shown in Table 2. 

The results show that sleep quality (sleep latency, sleep duration, sleep efficiency, and the PSQI subscales and total scores), and sleep hygiene (the SHPS subscales and total scores) of the participants without SI were significantly better than those of the participants with SI (*p* < 0.01). The participants without SI slept earlier and had lower ISI scores than those with SI (*p* < 0.01). The prevalence of all listed variables above or below the thresholds was higher in the participants with SI. In particular, the prevalence of those with SI whose PSQI and ISI scores exceeded the thresholds were 3.2 and 2.1 times that of those without SI, respectively. 

### 3.3. Correlational Analysis

Spearman’s correlations for all of the key variables in this study are displayed in Table 3. The results show that most of the key variables regarding sleep were significantly correlated with each other (all *p* < 0.05). The correlation between PSQI_Duration and PSQI_Medicine, PSQI_Duration and PSQI_Disturbance, and PSQI_Efficiency and PSQI_Medicine were not significant (*p* > 0.05). Furthermore, the association between sleep and depressive symptoms, and sleep and anxiety symptoms were all positively significant (*p* < 0.05). All the variables regarding sleep were significantly correlated with PSQI_SI (*p* < 0.05).

### 3.4. Hierarchical Linear Regression

The results of the hierarchical linear regression are shown in Table 4. Sex, sleeping schedules consistent with roommates, and relationship with roommates had significant effects on SI in the model (Step 1). As shown in Step 2, besides age and relationship with roommates, PSQI_Quality (β = 0.05, *p* = 0.03), PSQI_Duration (β = 0.07, *p* < 0.01), PSQI_Medicine (β = 0.12, *p* < 0.01), SHPS_Schedule (β = 0.09, *p* < 0.01), SHPS_Eating & drinking (β = 0.05, *p* = 0.03), SHPS_Environment (β = 0.05, *p* = 0.05), and ISI_Total (β = 0.12, *p* < 0.01) were positively associated with SI. Furthermore, sex and sleeping schedules consistent with roommates were no longer associated with SI in Step 2. As shown in Step 3, once depressive and anxiety symptoms were added to the model, shared bedroom at home (β = −0.05, *p* = 0.04), relationship with roommates (β = 0.05, *p* = 0.01), PSQI_Duration (β = 0.04, *p* = 0.05), PSQI_Medicine (β = 0.07, *p* < 0.01), PSQI_Daytime function (β = −0.09, *p* < 0.01), and SCL-90_Depression (β = 0.65, *p* < 0.05) were associated with SI. Age, PSQI_Quality, SHPS_Schedule, SHPS_Eating & drinking, SHPS_Environment, and ISI_Total were no longer associated with SI in Step 3. Therefore, sharing a bedroom at home and PSQI_Daytime function had changed from not being associated with SI to being negatively associated with SI. The statistical tests indicate that there is no collinearity problem among the independent variables (all VIF < 10).

## 4. Discussion

This study aimed to examine the association between sleep quality, sleep hygiene, insomnia symptoms, and SI, as well as to identify sleep-related variables that are associated with SI after controlling for depressive and anxiety symptoms. The results indicate that the sleep quality, sleep hygiene, and insomnia symptoms of the participants without SI were significantly better than those of the participants with SI. Most of the key variables regarding sleep, emotional symptoms, and SI were significantly correlated with each other, except for sleep duration and sleep medicine use. After adjusting the demonstrated variables, age, relationship with roommates, sleep quality, sleep duration, sleep medicine use, sleep schedules, eating and drinking habits before sleep, and insomnia symptoms were positively associated with SI. However, after controlling symptoms of depression and anxiety, only sharing a bedroom at home, relationship with roommates, sleep duration, sleep medicine use, and daytime function were significantly associated with SI.

In this sample, 54.18% of the participants used electronic equipment for more than 30 min during their bedtime, which may increase sleep latency and shorten REM sleep, thereby leading to a high state of arousal, a shorter sleep duration, and poor sleep quality [20]. Further, 77.39% had no experience with sharing a bedroom with peers at home, which may be quite different from the shared-dormitory experience that many students have at school. The sleeping schedules of 90.84% of the participants were inconsistent with their roommates, and 27.83% of these participants advised that this affected their sleep. Furthermore, 9.7% of the participants had PSQI scores higher than the threshold, and 29.5% had insomnia symptoms. However, only 3.49% of students sought treatment, such as psychological counseling, medical help, or both. This suggests that a large number of college students with sleep problems do not seek professional help.

Compared to the participants without SI, the participants with SI slept later, had longer sleep latency, shorter sleep duration, and lower sleep efficiency. The scores of the PSQI and subscales, the SHPS and subscales, and insomnia symptoms were all significantly higher in participants with SI than those without SI. Furthermore, their depressive and anxiety symptoms were also higher than those without SI. These results are consistent with those in previous studies, that is, individuals with SI may have more sleep difficulties and emotional problems than those without [3,21,22].

Most of the key variables regarding sleep quality, sleep hygiene, insomnia, emotional symptoms, and SI were positively associated with each other, except PSQI_Duration and PSQI_Medicine. This result is approximately the same as previous findings [6,7,23]. To better understand how sleep may be related to SI, we further conducted hierarchical linear regression. The results indicate that older age, poor relationship with roommates, poor sleep quality, short sleep duration, sleep medicine use, a poor sleep schedule, bad eating and drinking habits before sleep, bad sleep environment, and insomnia symptoms could positively predict SI. After controlling for depressive and anxiety symptoms, no experiences of sharing a bedroom at home, poor relationship with roommates, poor sleep quality, short sleep duration, sleep medicine use, better daytime function, or depressive symptoms were associated with SI. Furthermore, since anxiety symptoms are not significantly associated with SI in regression analysis, according to this study, anxiety symptoms might not need to be controlled when considering the association between sleep and SI. Despite our findings here, we submit that sleep has been found to be closely related to depression, anxiety and other mental health problems [24,25], and the vicious cycle of interaction between sleep and mental health problems might lead to increased suicidality, which we believe warrants further attention and investigation.

The results of previous studies regarding the association between sleep and SI remain inconsistent. Predictors about sleep quality for SI, such as sleep duration, sleep daytime function, and sleep medicine use have received less attention in the literature. A previous study has found that, compared to normal sleep duration (7–9 h), short sleep duration (≤6 h) was associated with the reporting of a prior suicide attempt; however, unlike what is found in the present study, longer sleep duration (≥10 h) was associated with a higher likelihood of SI [9]. Inconsistent with this study, previous research has not found that daytime function and sleep medicine use can be significantly associated with SI, independently [26]. Sleep latency was found to have a significant independent association with SI in another study [3], but not in the abovementioned study [26]. The existing data cannot support an in-depth interpretation of the differences between the above results.

To the best of our knowledge, the current study is the first to examine the relationship between sleep hygiene and SI in adults. Sleep hygiene, including a poor sleep schedule, bad eating and drinking habits before sleep, and a poor sleep environment were significantly associated with SI but were not associated with SI independently of depression and anxiety symptoms.

For insomnia symptoms, similar to most studies, this study finds that insomnia symptoms are associated with SI [6,7,27,28]. However, after adjusting for depressive and anxiety symptoms, insomnia symptoms did not show a significant association with SI, which was consistent with most previous studies [6,7,27,28]. However, these studies have found that the duration of insomnia symptoms may predict SI, and insomnia symptoms may predict SA and suicide deaths after controlling for depressive symptoms [27,28]. This further indicates that the association between insomnia and suicide is independent of depressive symptoms. Furthermore, inconsistent with this study, another previous study found that insomnia symptoms or sleep onset insomnia was associated with SI after controlling depressive symptoms [9]. Further studies are necessary to investigate the cause of such diverse results.

In addition, as most college students live in dormitories with roommates, this study investigated the association between college students’ sleeping habits and adaptation to their specific accommodation and SI. The results showed that a poor relationship with roommates or no experience of sharing a bedroom at home were positively associated with SI after adjusting for depressive and anxiety symptoms. Due to the results of previous studies indicating that poor relationship functioning is associated with greater self-reported arousal and insomnia severity [29], and that sleep onset latency mediates the association between depression and relationship functioning [30], we speculate that sleep may be the mediator of the above variables relationships with SI. Further studies are needed to verify this assumption.

The results of our study suggest that sleep quality should be included in the suicide risk assessment system. For example, sleep duration, sleep medicine use, and daytime function subscales of PSQI, and questions concerning sharing a bedroom with peers at home, and the relationship with roommates could be utilized, after eliminating depressive symptoms. Additionally, sleep intervention programs have certain advantages over suicide intervention by targeting other suicide risk factors, because poor sleep quality is more easily identified by family members and others around than other risk factors, and reducing suicide risk by seeking treatment for poor sleeping quality may be more acceptable for many individuals [23]. Therefore, treatment to improve sleep quality for suicidality prevention should be considered for individuals with SI and poor sleep quality. Furthermore, several psychotherapeutic approaches, such as cognitive-behavioral therapy [31], dialectical behavior therapy [32], and mindfulness meditation [33,34], which have been shown not only to have a positive effect on suicide risk but also to improve sleep quality, deserve more attention in research and interventions.

The following limitations of this study should be considered in interpreting our findings. First, the participants of this study were Chinese undergraduate students aged 18–26 years, affecting the generalizability of the results and making the findings most applicable to young Chinese people. Second, sleep data were collected through self-report questionnaires. Although a comparison of self-reported sleep time with actigraphy found high correlations between the two measures [35,36], self-reporting may still affect the objectivity and accuracy of the data. Third, we used a single item (Q15 of SCL-90: Thoughts of ending life) to assess SI, which may reduce the precision and comparability of the study. Fourth, the sleep duration subscale of PSQI we used only examined whether the sleep was sufficient, and did not consider the problem of excessive sleep. Only 13 participants in our sample slept longer than or equal to 10 h, so we did not investigate whether excessive sleep was associated with SI. Fifth, the existing data are insufficient to explain some results of this study in depth, such as why a better daytime function was associated with SI after adjusting for depressive and anxiety symptoms. Further research is needed to explain these results. Finally, this study was based on cross-sectional data. For future research, longitudinal and experimental research should be conducted to determine any causal relationships between relevant variables.

## 5. Conclusions

Sleep quality, sleep hygiene, and insomnia symptoms were significantly better for participants without SI than those with SI. Sleep, emotional symptoms, and SI were significantly correlated with each other, except for the correlation between sleep duration and sleep medicine use. The sharing of a bedroom at home, relationship with roommates, sleep duration, sleep medicine use, and daytime function were found to be associated with SI independently from depressive and anxiety symptoms. These results suggest that sleep quality should be included in the suicide risk assessment system. Sleep intervention programs may have certain advantages over suicide interventions, due to the ease of discoverability and modifiability, and reduced sense of shame. Therefore, we submit that the improvement of sleep quality warrants further attention from scholars as a means to achieve a reduction in suicidality.

## Figures and Tables

**Table 1:** Demographic characteristics of the participants (*N* = 2379).

Variables		*n*	*%*	Variables		*n*	*%*
Sex	Male	411	17.28%	Shares a bedroom with sibling at home	No sibling or does not share	1841	77.39%
	Female	1968	82.72%		Shares a bedroom	538	22.61%
Grade	Freshman	748	31.44%	Sleeping schedules consistent with roommates	Consistent	218	9.16%
	Sophomore	734	30.85%		Inconsistent but does not affect sleep	1499	63.01%
	Junior	543	22.82%		Inconsistent and does affect sleep	662	27.83%
	Senior	354	14.88%	Relationship with roommates	Good	2141	90.00%
School	National key university	871	36.61%		General	193	8.11%
	Municipal key university	1157	48.63%		Poor	45	1.89%
	General university	351	14.75%	Seek treatment for sleep problems	No	2296	96.51%
Electronic equipment use in bedtime	0 min	147	6.18%		Receive psychological counseling	35	1.47%
	<10 min	198	8.32%		Get medical attention	33	1.39%
	11–30 min	745	31.32%		Both	15	0.63%
	>30 min	1289	54.18%	Total		2379	100%

**Table 2:** Descriptive data and outcomes of Mann–Whitney U test.

	All Participants (*N* = 2379)		Participants with SI (*n* = 327)		Participants without SI (*n* = 2052)		
Variables	Median (IQR)	*n (%)* (above or below the Thresholds)	Median (IQR)	*n (%)* (above or below the Thresholds)	Median (IQR)	*n (%)* (above or below the Thresholds)	*p* of Mann-Whitney U Test
Bedtime (hh:mm)	23:30 (21:30–01:30)	1499 (63.1%)	24:00 (21:30–01:30)	231 (70.6%)	23:30 (21:30–01:30)	1268 (61.8%)	<0.01
Sleep latency (hh:mm)	00:15 (00–01:00)	738 (31%)	00:20 (0–01:00)	143 (43.7%)	00:15 (0–01:00)	595 (29%)	<0.01
Sleep duration (h)	7 (5–9)	1099 (46.1%)	7 (4.5–8.5)	162 (49.5%)	7 (5–9)	697 (34%)	<0.01
Sleep efficiency (%)	92.31 (1.5–5.5)	554 (23.3%)	88.89 (0.64–1.15)	95 (29.1%)	92.86 (0.69–1.14)	459 (22.4%)	<0.01
PSQI_Quality	0 (0–0)		0 (0–2.5)		0 (0–0)		<0.01
PSQI_Latency	1 (0–2.5)		1 (0–5)		1 (0–2.5)		<0.01
PSQI_Duration	1 (0–2.5)		1 (1–1)		1 (0–2.5)		<0.01
PSQI_Efficiency	0 (0–0)		0 (0–2.5)		0 (0–0)		<0.01
PSQI_Disturbance	1 (0–2.5)		1 (0–2.5)		1 (0–2.5)		<0.01
PSQI_Medicine	0 (0–0)		0 (0–0)		0 (0–0)		<0.01
PSQI_Daytime function	1 (0–5)		1 (0–3.5)		1 (0–5)		<0.01
PSQI_Total	3 (0–9.5)	230 (9.7%)	5 (0–10.5)	78 (23.9%)	3 (0–9.5)	152 (7.4%)	<0.01
SHPS_Arousal behaviors	24 (6.5–42.5)		27 (11–43)		24 (8–40)		<0.01
SHPS_Schedule	21 (6–38)		26 (10–42)		21 (5–37)		<0.01
SHPS_Eating & drinking	11 (1.5–21.5)		13 (1–25)		11 (1.5–21.5)		<0.01
SHPS_Environment	17 (0–37)		21 (0–43.5)		16 (0–37)		<0.01
SHPS_Total	75 (29.5–121.5)		87 (40–136)		74 (30–118)		<0.01
ISI_Total	5 (0–17)	702 (29.5%)	8 (0–22.5)	177 (54.1%)	4 (0–17)	525 (25.6%)	<0.01
SCL-90_Depression	1.38 (0–3.19)	547 (23.0%)	2.46 (0.31–4.62)	241 (73.7%)	1.27 (0.15–2.62)	306 (14.9%)	<0.01
SCL-90_Anxiety	1.20 (0–2.75)	508 (17.1%)	2.10 (0.1–4.1)	288 (88.3%)	1.20 (0.25–2.25)	220 (10.7%)	<0.01

IQR, interquartile range; SI, suicidal ideation; PSQI, Pittsburgh Sleep Quality Index; SHPS, Sleep Hygiene Practice Scale; ISI, Insomnia Severity Index; SCL-90, tSymptom Checklist 90.

**Table 3:** Correlations of all the key variables (*N* = 2379).

	Variables	1	2	3	4	5	6	7	8	9	10	11	12	13	14	15	16
1	PSQI_Quality																
2	PSQI_Latency	0.36 **															
3	PSQI_Duration	0.17 **	0.08 **														
4	PSQI_Efficiency	0.15 **	0.30 **	0.25 **													
5	PSQI_Disturbance	0.22 **	0.27 **	0.03	0.09 **												
6	PSQI_Medicine	0.14 **	0.15 **	−0.003	0.04	0.09 **											
7	PSQI_Daytime function	0.30 **	0.20 **	0.20 **	0.09 **	0.24 **	0.06 **										
8	PSQI_Total	0.55 **	0.64 **	0.49 **	0.48 **	0.52 **	0.19 **	0.66 **									
9	SHPS_Arousal behaviors	0.18 **	0.20 **	0.10 **	0.11 **	0.14 **	0.06 **	0.37 **	0.34 **								
10	SHPS_Schedule	0.33 **	0.43 **	0.16 **	0.15 **	0.33 **	0.13 **	0.36 **	0.52 **	0.41 **							
11	SHPS_Eating & drinking	0.10 **	0.11 **	0.11 **	0.05 **	0.11 **	0.07 **	0.26 **	0.24 **	0.40 **	0.42 **						
12	SHPS_Environment	0.25 **	0.26 **	0.06 **	0.07 **	0.24 **	0.07 **	0.32 **	0.36 **	0.34 **	0.50 **	0.39 **					
13	SHPS_Total	0.31 **	0.35 **	0.15 **	0.14 **	0.28 **	0.11 **	0.44 **	0.51 **	0.71 **	0.79 **	0.64 **	0.78 **				
14	ISI_Total	0.52 **	0.45 **	0.23 **	0.19 **	0.34 **	0.15 **	0.46 **	0.63 **	0.34 **	0.59 **	0.28 **	0.47 **	0.57 **			
15	SCL-90_Depression	0.33 **	0.23 **	0.19 **	0.08 **	0.27 **	0.12 **	0.46 **	0.47 **	0.36 **	0.47 **	0.30 **	0.42 **	0.53 **	0.52 **		
16	SCL-90_Anxiety	0.27 **	0.21 **	0.15 **	0.06 **	0.27 **	0.10 **	0.38 **	0.40 **	0.29 **	0.47 **	0.26 **	0.41 **	0.49 **	0.49 **	0.81 **	
17	SCL-90_SI	0.21 **	0.13 **	0.14 **	0.07 **	0.12 **	0.12 **	0.18 **	0.23 **	0.17 **	0.24 **	0.16 **	0.21 **	0.27 **	0.25 **	0.47 **	0.41 **

** *p* < 0.01; SI, suicidal ideation; PSQI, Pittsburgh Sleep Quality Index; SHPS, Sleep Hygiene Practice Scale; ISI, Insomnia Severity Index; SCL-90, Symptom Checklist 90.

**Table 4:** Hierarchical linear regression models associating sleep with SI (*N* = 2379).

Variables	*B*	*SE*	β	*t*	*p*	R2	Adjusted R2	R2 Change	*F*	*p*
**Step 1**						0.04	0.04	0.04	21.34	<0.01
Age	−0.02	0.01	−0.04	−1.76	0.08					
Sex	0.07	0.03	0.04	2.03	**0.04**					
Shares a bedroom at home	−0.04	0.03	−0.03	−1.40	0.16					
Sleeping schedules consistent with roommates	0.07	0.02	0.06	2.92	**<0.01**					
Relationship with roommates	0.30	0.03	0.18	8.71	**<0.01**					
**Step 2**						0.14	0.14	0.10	23.32	<0.01
Age	−0.02	0.01	−0.04	−2.02	**0.04**					
Sex	0.03	0.03	0.02	1.07	0.29					
Shares a bedroom at home	−0.05	0.03	−0.03	−1.74	0.08					
Sleeping schedules consistent with roommates	−0.02	0.02	−0.02	−1.07	0.28					
Relationship with roommates	0.19	0.03	0.12	5.82	**<0.01**					
PSQI_Quality	0.07	0.03	0.05	2.21	**0.03**					
PSQI_Latency	−0.02	0.02	−0.03	−1.15	0.25					
PSQI_Duration	0.07	0.02	0.07	3.22	**<0.01**					
PSQI_Efficiency	0.01	0.02	0.01	0.34	0.74					
PSQI_Disturbance	−0.01	0.03	−0.01	−0.26	0.79					
PSQI_Medicine	0.33	0.05	0.12	6.18	**<0.01**					
PSQI_Daytime	0.02	0.02	0.02	0.91	0.37					
SHPS_Arousal behaviors	0.00	0.00	0.01	0.65	0.52					
SHPS_Schedule	0.01	0.00	0.09	3.08	**<0.01**					
SHPS_Eating & drinking	0.01	0.00	0.05	2.12	**0.03**					
SHPS_Environment	0.00	0.00	0.05	1.96	**0.05**					
ISI_Total	0.02	0.00	0.12	3.87	**<0.01**					
**Step 3**						0.38	0.38	0.24	76.26	<0.01
Age	−0.01	0.01	−0.01	−0.68	0.50					
Sex	0.01	0.03	0.01	0.51	0.61					
Shares a bedroom at home	−0.05	0.02	−0.03	−2.07	**0.04**					
Sleeping schedules consistent with roommates	0.00	0.02	0.00	0.05	0.96					
Relationship with roommates	0.08	0.03	0.05	2.79	**0.01**					
PSQI_Quality	0.02	0.03	0.01	0.58	0.56					
PSQI_Latency	0.01	0.02	0.01	0.60	0.55					
PSQI_Duration	0.04	0.02	0.04	1.99	**0.05**					
PSQI_Efficiency	0.02	0.02	0.02	0.87	0.38					
PSQI_Disturbance	−0.03	0.02	-0.02	-1.38	0.17					
PSQI_ Medicine use	0.19	0.05	0.07	4.23	**<0.01**					
PSQI_ Daytime	−0.07	0.02	−0.09	−4.51	**<0.01**					
SHPS_Arousal behaviors	0.00	0.00	−0.03	−1.55	0.12					
SHPS_Schedule	0.00	0.00	−0.01	−0.39	0.69					
SHPS_Eating and drinking	0.00	0.00	0.02	1.28	0.20					
SHPS_Environment	0.00	0.00	0.00	−0.09	0.93					
ISI_Total	0.00	0.00	−0.01	−0.49	0.63					
SCL-90_Depression	0.61	0.03	0.65	20.37	**<0.01**					
SCL-90_Anxiety	−0.04	0.03	−0.04	−1.24	0.22					

Bold values denote statistical significance; SI, suicidal ideation; PSQI, Pittsburgh Sleep Quality Index; SHPS, Sleep Hygiene Practice Scale; ISI, Insomnia Severity Index; SCL-90, Symptom Checklist 90.

## Data Availability

The datasets generated during the current study are not publicly available but are available from the corresponding author on reasonable request.
